# The Veterans Choice Act and Technical Efficiency of Veterans Affairs (VA) Hospitals

**DOI:** 10.3390/healthcare10061101

**Published:** 2022-06-13

**Authors:** Dongjin Oh, Keon-Hyung Lee, Jongsun Park

**Affiliations:** 1 Graduate School of Defense Management, Korea National Defense University, Nonsan 33021, Korea; dongjinoh64@gmail.com; 2 Askew School of Public Administration and Policy, Florida State University, Tallahassee, FL 32306, USA; klee2@fsu.edu; 3 Department of Public Administration, Gachon University, Seongnam 13120, Korea

**Keywords:** veterans, public health, health policy, technical efficiency, VA hospital

## Abstract

The Veterans Health Administration (VHA), responsible for providing 9 million veterans with quality healthcare, is not insulated from concerns about efficiency. In the aftermath of the Veterans Affairs (VA) hospital scandal in 2014, Congress passed the Veterans Choice Act of 2014, which allows eligible veterans to use non-VA hospitals instead of VA hospitals. After analyzing 118 or 119 VA hospitals each year from 2012 through 2017 in the U.S, this paper evaluates the efficiency scores of VA hospitals and examines how the 2014 Act has influenced their technical efficiency over time. Slack analysis shows that inefficient VA hospitals can improve efficiency by reallocating input resources, and regression analysis demonstrates that the overall technical efficiency of VA hospitals decreased by 0.164 after the implementation of the Act. This means that as more veterans used non-VA hospitals under the 2014 Act, the technical efficiency of VA hospitals decreased considerably. Given that a substantial portion of veterans’ demands for healthcare transferred out to non-VA hospitals, the VHA should evaluate whether the current capacity of VA hospitals is appropriate and try to reduce wasted input resources to improve efficiency.

## 1. Introduction

The Veterans Health Administration (VHA), under the Department of Veterans Affairs (VA), is not insulated from concerns about efficiency. The VHA, responsible for providing 9 million veterans with quality healthcare, has the largest integrated healthcare delivery system in the United States [1]. According to the 2020 budget, the Department of Veterans Affairs (VA) requested USD 84.1 billion in discretionary funding for veterans’ medical care, which will support 348,389 medical care FTEs [2]. These enormous VA resources should be effectively managed in ways that meet the healthcare needs of veterans while controlling expenditures. To achieve this, the VHA must probe the current healthcare delivery systems to identify and improve any inefficient areas. Although numerous scholars have examined hospital efficiency, only a few studies have done so specifically for VA hospitals [3,4,5,6].

Moreover, the Veterans Access, Choice, and Accountability Act of 2014—often referred to as the Veterans Choice Act—may influence VA hospital utilization. In 2014, a whistleblower disclosed that many veterans died without receiving needed treatments due to extended wait times. VA hospital executives and employees failed to meet the goal of a 14-day wait time (even manipulating wait time records) and, nonetheless, received USD 142 million in bonuses for the fiscal year 2014 [7]. As a reaction to the scandal, Congress passed the Veterans Choice Act on 7 August 2014. The Veterans Choice Act required the Department of Veterans Affairs (VA) to implement the Veterans Choice Program (VCP) that allows a veteran to use non-VA hospitals if he/she lives 40 miles or more from a VA healthcare facility or has to wait more than 30 days to receive needed healthcare [8]. Under the VCP, eligible veterans can choose to receive medical care from community care providers (VCP providers) that have VA-required credentials or licenses [8]. The total number of authorizations of care for the VCP between November 2014 and August 2018 was 5,909,713 [8]. The VA appropriated USD 8.2 billion for community care in the fiscal year 2014, which increased to USD 14.9 billion in the fiscal year 2018 [9]. However, there has been little research on the potential impact of the Act on VA hospital utilization. To fill the academic gap, this study analyzes the efficiency of VA hospitals before and after the Act of 2014, taking a two-step approach: (1) evaluating VA hospital efficiency scores; and (2) investigating the determinants that affect hospital efficiency. The results show that the overall efficiency of VA hospitals substantially decreased after the implementation of the Veterans Choice Act of 2014 and that an increase in the number of veterans using non-VA hospitals negatively influenced the efficiency of VA hospitals.

## 2. Theoretical Explanations of Technical Efficiency Determinants

Technical efficiency refers to achieving maximum production by using given input resources while minimizing wasted resources [10]. Technical efficiency, calculated as a ratio of output to input, is used to identify the efficiency frontier representing a relative best performance that all organizations in a sample can achieve [11]. If a VA hospital is technically efficient, it should be on the efficiency frontier. This can be utilized as a benchmark to identify best practices in VA hospitals. In this paper, we use the concepts of technical efficiency and frontier analysis to examine how the efficiency of VA hospitals changed before and after the Veterans Choice Act of 2014. Numerous scholars have used the concept of technical efficiency to measure performance and identify inefficient areas of hospitals in either the private or public sectors [3,4,5,6,12,13,14]. However, there has been little research on the determinants of VA hospital technical efficiency. Thus, this study contributes to the knowledge about VA hospitals by analyzing the relative technical efficiencies of VA hospitals and identifying distinctive determinants of efficiency over the period of 2012 to 2017.

### 2.1. Political Constraints on the Department of Veterans Affairs

Public organizations are open systems that constantly interact with the external environment, which can significantly influence their performance [15]. Wilson [16] demonstrated that the inefficiency and inflexibility of government agencies are caused by political constraints. The Department of Veterans Affairs (VA) is in client politics, where most of the benefits from the VA are distributed to a relatively small interest group—veterans—while the costs for the benefits are shared by most taxpayers [16]. Since veterans’ benefits are significant and the cost per capita is small, they have sufficient cause to organize themselves and impose pressure on Congress and the VA to create policies in their interest [16].

Furthermore, according to the social construction theory, veterans belong to an “advantaged group” that has a high level of political power and public support as a deserving group, resulting in substantial welfare policy benefits [17]. Politicians and policymakers tend to enact policies in favor of veterans to earn substantial political points [17]. Moreover, since politicians want to effect quick policy achievements in order to be re-elected, the VA tends to experience frequent changes in veteran policies that management officials in the VA cannot control. 

Under the Veterans Choice Act of 2014, a veteran eligible for the Veterans Choice Program (VCP) can choose to receive medical service either from a VA hospital or a community care provider. Thus, the implementation of the VCP likely led more unserved veterans to receive timely healthcare. Since the implementation of the VCP, the number of veterans who have used the VCP is estimated to be 1.9 million (24 million appointments) [18]. While increased competition in the healthcare market can compel private hospitals to be more efficient [12,19], VA hospitals may not be able to flexibly adapt to the decreasing healthcare demands of veterans by correspondingly reducing input resources, such as operating costs, directly hired physicians, nurses, or other FTEs. This is because private organizations can utilize resources in ways that maximize productivity and profits by adjusting input and output resources depending on the market environment, but public organizations cannot adjust as quickly because of political constraints that require them to consider social values such as equity, accountability and responsiveness to constituencies, and fiscal integrity [16]. Thus, because of political constraints surrounding the VA, we expect that VA hospitals will not be able to reduce input resources following decreased demands after 2014, which will lead to weakened efficiency.

**Hypothesis** **1.** *The technical efficiency of VA hospitals will decrease following the implementation of the Veterans Choice Act of 2014*.

### 2.2. Task of VA Hospital

The VHA is not only the largest fully integrated healthcare system but also the largest provider of medical education for junior physicians, as well as medical students [20]. The VHA is estimated to train about 70 percent of U.S. physicians, medical students, and other medical trainees [21], and 64 VA hospitals belong to the Council of Teaching Hospitals (COTH). Since teaching hospitals must have enough resources to operate medical education programs while satisfying quality healthcare standards for patients, the hospitals require more input resources than non-teaching hospitals, which leads to technical inefficiency [13,22].

**Hypothesis** **2.** *Teaching VA hospitals will be less technically efficient than non-teaching VA hospitals.*

Diagnosis-related groups (DRGs) classify acute patients into homogeneous groups based on similar ICD-9-CM (International Classification Diseases, Ninth Revision, Clinical Modification) diseases [23], which are used for the Medicare prospective payment system (PPS) [24]. Patients within the same DRG are assumed to receive similar levels of clinical treatments, which leads to similar consumption of hospital resources. The case mix index (CMI), based on Medicare DRG, reflects the severity of patients’ conditions within a hospital. CMI-adjustment provides rough comparisons of cost and resource consumption among hospitals [25]. A higher CMI is associated with an increased risk of mortality and difficulty of treatment, which results in greater consumption of resources. Thus, a high CMI is a cost driver for hospitals and negatively impacts efficiency [26]. However, VA hospitals have not adopted CMI because they do not treat Medicare patients. Instead of the CMI, the VHA assigns one of three complexity levels to each VA hospital, depending on patient risks, teaching and research activities, the number of medical employees, and levels of intensive care units (ICUs). A VA hospital with a complexity level of 1 is more likely to treat severe patients than a VA hospital with a complexity level of 2 or 3.

**Hypothesis** **3.** *A VA hospital with complexity level 1 is more likely to be less technically efficient than a VA hospital with complexity level 2 or 3.*

## 3. Materials and Methods

### 3.1. Sample

We collected data from three sources. First, we used the American Hospital Association (AHA) annual surveys from 2012 through 2017 (excluding 2014) to obtain input and output data in order to measure the technical efficiency and COTH membership of VA hospitals. Since the Veterans Choice Act of 2014 became effective in August 2014, we treated 2014 as a washout period and excluded samples of 2014 in the analysis. Second, we used the U.S. Census Fact Finder to gather data on veterans’ educational attainment, unemployment rate, poverty rate, and disability rate in the county where a VA hospital is located. Finally, we obtained the complexity level data on each VA hospital, data on the numbers of veteran patients visiting VA hospitals, and the number of veteran patients using a community care provider from 2015 through 2017 from the VHA through an information request under the Freedom of Information Act (FOIA). We combined the data from the three sources into panel data and excluded observations with missing values. The final working samples were 118 or 119 hospitals each year.

### 3.2. Measurement of Technical Efficiency of VA Hospitals

We measured the technical efficiencies of 118 or 119 VA hospitals separately from 2012 through 2017 by years (excluding 2014) in the United States using the Data Envelopment Analysis (DEA) to identify the most efficient VA hospitals. We applied an input-oriented version of the DEA to identify a minimum level of input resources to produce the observed output because hospitals have more control over input resources than output. Moreover, we chose the variable-return-to-scale (VRS), which assumes a nonlinear relationship between inputs and outputs. The technical efficiency scores fall between 0 and 1, meaning 1 is the most efficient, and 0 is the least efficient. A hospital achieving 1 should be on the efficiency frontier.

We chose three types of input and four types of output variables commonly used in previous research to estimate hospital efficiency [5,6,27,28]. The three types of input variables are (1) operating expenses, (2) the number of hospital beds, and (3) the number of full-time equivalent (FTE) employees, excluding FTE trainees. The four types of output variables are (1) inpatient days, (2) the number of surgical operations, (3) the number of outpatient visits, and (4) the number of FTE trainees. We included FTE trainees as an output variable because the VHA is the largest provider of medical training in the United States and one of its primary missions is medical research and education [20]. The number of FTE trainees is the sum of FTE medical/dental residents and interns, as well as other FTE professional trainees. Table 1 shows the means and standard deviations of the input and output variables. 

### 3.3. Measurement of Determinants of Technical Efficiency

#### 3.3.1. Independent Variables

The effect of the “Veterans Choice Act” was measured as binary. Since the Act became effective in August 2014, veterans’ demands for healthcare in VA hospitals would likely change following its implementation. We excluded 2014 as a washout period and measured the effect of the Act as 1 if the year is equal to or greater than 2015, or 0 otherwise. Moreover, we divided the effect of the Act into each year (2015, 2016, and 2017) to see whether the Act has had a consistent effect since its implementation. The variables were measured as binary: 1 if the year is 2015, 2016, and 2017, or 0 otherwise. Those variables substitute for the “Veterans Choice Act” in Model 2. 

In addition, we measured the percentage of veterans who used the Veterans Choice Programs (VCPs) at the VA hospital level from 2015 through 2017 to examine whether changes in the percentage have had an impact on the efficiency levels of VA hospitals. The percentage of veterans using the VCP at the VA hospital level was measured as the total number of veterans using the VCP divided by the total number of veterans who likely visit each VA hospital. This variable substitutes for the “Veterans Choice Act” in Model 3.

The Council of Teaching Hospitals and Health Systems (COTH) consists of 400 major teaching hospitals in the United States and Canada [29]. “Teaching Status” is measured as binary: 1 if a VA hospital has a COTH membership, or 0 otherwise. “Complexity” was measured as 3 categorical variables: 1 if the complexity level is 1, 2, or 3, respectively, or 0 otherwise. We used complexity 1 as a reference group. 

#### 3.3.2. Control Variables

At the hospital level, we controlled for hospital size measured by the number of beds in a hospital. Furthermore, we added attributes of the county where a VA hospital was located: the number of veteran residents, the percentage of veterans having a bachelor’s degree or above, veterans’ unemployment rate, the percentage of veterans having any disability, and the percentage of veterans below the Federal Poverty Level (FPL) in the last 12 months. We controlled for those county attributes because veterans who are of low socioeconomic status and have a disability are more likely to rely on VA healthcare [30], which could affect the efficiency of a VA hospital. At the state level, we controlled for Medicaid expansion status because veterans living in Medicaid states are less likely to use VA hospitals than those living in non-expansion states [31]. Moreover, we added year ordinal variables to control the year effect in the model: 1 for 2012, 2 for 2013, …, and 6 for 2017. Table 2 presents the characteristics of the samples.

#### 3.3.3. Statistical Analysis

This study analyzed the efficiency of VA hospitals before and after 2014, taking a two-step approach. In the first stage, we ran a DEA analysis to estimate the technical efficiency of VA hospitals each year. In the second stage, we investigated the factors that have a significant effect on the technical efficiency of VA hospitals. Given that the dependent variable (technical efficiency scores) is limited to values between 0 and 1, and that the samples are 4-year panel data of VA hospitals, we used a cross-sectional time-series tobit regression of Stata Version 14. The tobit model, also called a truncated/censored regression model, strives to examine linear relationships between variables when there is either left or right censored distribution in the dependent variable. In our model, tobit regression was used because the technique is appropriate for censored dependent variables because the DEA scores range from 0 to 1 (right censored at 1). Numerous previous research studies on hospital efficiency widely used the tobit model [27,28]. The estimated technical efficiency in the first stage was used as a dependent variable in the second stage, which was censored at a value of 1. We integrated all variables into the following regression equation: “α” indicates an intercept of the regression, and “$\beta_{i}$” means the coefficients of the independent or control variables. Additionally, we conducted all regression assumption diagnostic tests such as non-perfect collinearity, zero-conditional means, and homoscedasticity tests [32]. This model did not violate the assumptions. 

First, to examine the perfect collinearity problem, we conducted Variance Inflation Factor (VIF) test. High VIF values cause a high variance of the estimators, leading to imprecise estimators. The tolerance value (defined as 1/VIF) is 0.1, which is widely used to test the collinearity assumption. A tolerance value smaller than 0.1 implies that the variable can be expressed by linear combinations of the other independent variables [32]. The lowest tolerance value was 0.129 of the “Choice Act”. Thus, this regression model satisfied the non-perfect collinearity assumption. Second, the zero-conditional means assumption can be examined by the specification error test. We carried out the Ramsey regression specification error test (RESET) for omitted variables. The null hypothesis is no omitted variables. Since the *p*-value is 0.2589 (*F* value = 1.34), we failed to reject the null hypothesis. Thus, this regression model does not suffer from specification errors. Third, homoskedasticity was examined by the Breusch–Pagan test. The null hypothesis is homoskedasticity (constant variance). Since the *p*-value is 0.6980, we failed to reject the null hypothesis. Thus, this regression model satisfied the homoskedasticity assumption.

(1) $$\begin{array}{l} \mathrm{Technical}\;\mathrm{Efficiency}\;=\alpha +\beta_{1}Choice\;Act+\beta_{2}COTH+\beta_{3}Complexity\;2+\\ \beta_{4}Complexity\;3+\beta_{5}Beds+\beta_{6}Veteran\;residents+\beta_{7}\;Education+\beta_{8}\;Poverty+\\ \beta_{9}Unemployment+\beta_{10}\;Disability+\beta_{11\;}Medicaid+\beta_{12\;}Year+u \end{array}$$

This research aims to investigate how the implementation of the Veterans Choice Act influenced VA hospital technical efficiency. To answer the research question, we used three models. In Model 1, we examined whether the technical efficiency of VA hospitals significantly decreased after the Act by using a dummy variable, “Choice Act” (i.e., 1 if year > 2014, 0 otherwise). In Model 2, we substituted the dummy variable with “Veterans Choice Act in 2015, 2016, and 2017” to see whether the effect of the Act is statistically significant in each year following its implementation. The year variables were measured as a binary: 1 if the year is 2015, 2016, and 2017, respectively, or 0 otherwise. The reference group is comprised of the samples in 2012 and 2013 (before the implementation of the Veterans Choice Act). In Model 3, we investigated how the percentage of veterans using the VCP influenced the efficiency of VA hospitals from 2015 to 2017.

## 4. Results

### 4.1. Stage 1: Estimation of Technical Efficiency Scores

We ran the Data Envelopment Analysis (DEA) to estimate the technical efficiency scores of the VA hospitals each year. Table 3 shows the results of the DEA analysis. The average efficiency scores, which were 0.8 in 2012 and 0.84 in 2013, decreased to 0.79 in 2015 and 0.72 in 2016 but recovered to 0.83 in 2017. The results alone could not reveal whether the VA hospitals’ decrease in efficiency scores was caused by the Veterans Choice Act or other factors. Thus, we conducted a regression analysis to examine the effect of the Act on the technical efficiency of VA hospitals. 

In Table 3, we also present the average input slack among inefficient VA hospitals in each year. The term slack means the magnitude of relative inefficiency caused by overused inputs or underproduced outputs, given an efficiency frontier [5,11]. An input slack is the difference between actual input and target input. A VA hospital must reduce input slack (overused input resources) to be on an efficiency frontier (efficiency score 1). The average overused operating expenses were at a minimum of USD 34.6 million in 2015 and at a maximum of USD 86.9 million in 2016. The excess number of full-time equivalent (FTE) employees was lowest at 331 in 2012 and highest at 733 in 2015. In addition, VA hospitals must reduce excess beds, on average between 30 (2017) and 69 (2015), to be more efficient. The result of the slack analysis demonstrates that VA hospitals can save substantial input resources without compromising the observed output levels. 

### 4.2. Stage 2: Regression Analysis of Technical Efficiency

We conducted cross-sectional time-series tobit regressions to examine the effects of the Veterans Choice Act and other factors on the changes in VA hospital technical efficiency scores. The regression models were statistically significant based on Wald chi-squared test. Table 4 shows the results of the regression analysis. 

First, the implementation of the Veterans Choice Act from 2015 to 2017 was associated with a decrease in technical efficiency in VA hospitals: the overall technical efficiency decreased by 0.164 after the implementation of the Act. In Model 2, the effect of the Act was consistently significant from 2015 to 2017. Compared to the VA hospitals’ efficiency scores in 2012 and 2013, before the Act, the scores of VA hospitals decreased by, on average, 0.193 in 2015, 0.328 in 2016, and 0.261 in 2017. Moreover, in Model 3, we investigated how the percentage of veterans using the VCP influenced the efficiency of VA hospitals from 2015 to 2017. As the percentage of veteran patients who used the VCP increased, the efficiency scores of VA hospitals decreased. This was an increase of one percent in the number of veterans using the VCP which led to, on average, a decrease of 0.005 in the efficiency score. This finding suggests that as a substantial number of veterans eligible for the VCP visited non-VA hospitals, utilization of VA hospitals substantially decreased more than before the Act, but input resources were not reduced correspondingly with the decreased demands of veterans for VA healthcare. 

Second, VA hospitals dealing with more severe patients were less efficient. VA hospitals with complexity levels of 2 and 3 were more efficient than VA hospitals with complexity level 1, a finding which was robust throughout the 3 models. This result implies that since VA hospitals with complexity level 1 are likely to deal with high-risk mortality and provide complex treatments to high-risk patients, they should consume more input resources than VA hospitals with complexity levels 2 or 3. This finding was consistent with previous research that shows that a high Case Mix Index is the main cost driver for a hospital [26] and that a higher proportion of less complex patients contributes to the higher efficiency of a hospital [14]. 

Third, the effect of teaching status measured by COTH membership was statistically insignificant on the technical efficiency of VA hospitals. We conducted a sensitivity analysis on technical efficiency, measured without the FTE trainees as an output resource, but the effect of COTH membership was still insignificant. This result was inconsistent with the previous research arguing that teaching status has a negative significant effect on the efficiency of hospitals in the non-government sector [13,22,33]. The discrepancy in the effect of teaching status might derive from a difference in hospital ownership because ownership—profit vs. nonprofit, for example—is a strong predictor of efficiency [22]. VA hospitals are interwoven within the Veteran Integrated Service Networks (VISNs) and provide healthcare only to eligible veterans, not to Medicaid and Medicare patients. Furthermore, VA hospitals are the largest providers of medical training in the Unities States and account for 14% of total COTH member hospitals [30]. 

Fourth, the effects of environmental variables such as county-level attributes and Medicaid expansion were uniformly insignificant, which was consistent across the three models. The insignificant effect of the environmental variables implies that hospital efficiency is not a matter of the environmental and demographic characteristics of the county in which the hospital is located, but a matter of management of hospital resources. Thus, hospital managers should identify inefficient areas of management (i.e., input slacks) and strive to minimize them to improve hospital efficiency. 

## 5. Conclusions and Implications

The findings of this study suggest that implementing the Veterans Choice Act without reallocating input resources did exacerbate the inefficiency of VA hospitals. Despite about 1.9 million veterans having visited community care providers instead of VA hospitals since the inception of the VCP [18], the average operating expense per hospital increased from USD 135 million in 2013 to USD 168 million in 2017 (See Table 1). The VCP contributed to more accessible healthcare services for veterans, but a substantial decrease in veterans’ use of VA hospitals led to significant inefficiency across VA hospitals. Thus, the VHA should evaluate whether the current capacity of VA hospitals is appropriate and try to reduce wasted input resources to improve efficiency. 

In addition, the VHA should examine the relative costs between two options: purchasing medical services from community care providers (market) or securing the same level of accessibility by expanding the capacity of VA hospitals. According to transaction cost theory, a decision between producing services within an organization and contracting out services to a third party depends on the relative costs between the internal and external transactions [34]. After evaluating the relative costs between the two options, the VHA should determine whether to continue providing community care benefits or not. Additionally, the VHA should consider closing VA hospitals with low efficiency and high input slack and instead refer veterans’ medical demands to community providers. 

At the VA hospital level, managers should try to learn the best practices of efficient VA hospitals. Although all VA hospitals face political constraints that impede managers’ ability to utilize resources in ways that maximize efficiency, some VA hospitals are at an efficiency frontier and some are not. Managers working in inefficient VA hospitals can improve the efficiency of their VA hospitals by adopting the best practices of efficient VA hospitals. In addition, given that VA hospitals dealing with more complex cases are likely to be less efficient, medium- and small-sized VA hospitals can improve their efficiency by referring complex patients to specialized VA hospitals. 

## Figures and Tables

**Table 1:** Descriptive Statistics of Input and Output Variables.

Variable	2012 (n = 119) Mean (SD)	2013 (n = 119) Mean (SD)	2015 (n = 118) Mean (SD)	2016 (n = 119) Mean (SD)	2017 (n = 119) Mean (SD)
Inputs					
Expenses (Millions)	USD 135 (USD 95)	USD 142 (USD 101)	USD 180 (USD 136)	USD 161 (USD 73)	USD 168 (USD 80)
FTEs	1896 (1235)	1806 (1222)	1990 (1225)	2274 (1614)	2110 (1397)
Beds	264 (223)	264 (225)	275 (229)	269 (221)	264 (218)
Outputs					
Inpatient days	63,176 (53,460)	64,174 (53,526)	67,329 (55,289)	68,199 (63,669)	69,468 (68,965)
Surgeries	3498 (2083)	3277 (2004)	4144 (2439)	3584 (2186)	3904 (3036)
Outpatient visits	506,558 (33,8507)	565,736 (356,737)	637,421 (355,454)	654,833 (440,230)	643,762 (532,547)
FTE trainees	59 (85)	184 (220)	77 (115)	83 (92)	70 (160)

Note. Observations in 2014 were excluded because 2014 is regarded as a washout period.

**Table 2:** Characteristics of Samples by Variables.

Variables	2012 (n = 119)	2013 (n = 119)	2015 (n = 118)	2016 (n = 119)	2017 (n = 119)
Choice Act	-	-	118	119	119
Veterans using Choice program (%)	-	-	2.13	10.55	12.63
VA hospitals with COTH Membership	77	78	76	79	79
VA hospitals with Complexity Level 1	87	86	86	87	87
VA hospitals with Complexity Level 2	13	14	14	14	14
VA hospitals with Complexity Level 3	19	19	18	18	18
Average Number of Hospital Beds	264	264	278	269	264
Veteran Residents in county (1,000 s)	50.2	45.8	44.3	43	42.5
Veterans with Bachelor or above in county (%)	27.4	27.7	28.9	29.6	29.8
Veteran Poverty rate in county (%)	8.2	8.2	8.2	7.8	8.0
Veteran Unemployment rate in county (%)	8.1	7.9	5.6	5.3	4.6
Veterans with Any Disability in county (%)	26.8	28.8	29.4	29.7	30.0
VA hospitals in Medicaid Expansion State	0	0	76	79	79

**Table 3:** Technical Efficiency Scores and Input Slack Analysis.

Results	2012 (n = 119)	2013 (n = 119)	2015 (n = 118)	2016 (n = 119)	2017 (n = 119)
Technical Efficiency					
Average Efficiency Score	0.80	0.84	0.79	0.72	0.83
Standard Deviation of Score	0.14	0.16	0.13	0.15	0.14
Minimum Efficiency Score	0.36	0.38	0.49	0.37	0.38
Maximum Efficiency Score	1	1	1	1	1
Number of the Most Efficient VA hospitals	22	36	21	16	31
Number of Inefficient VA hospitals	97	83	97	103	88
Average Slack of Input Resources					
Expenses (Millions)	58.7	37.4	34.6	86.9	69.9
FTEs excluding trainees	331	609	733	468	294
Beds	49.4	67	69	56	30

**Table 4:** Results from cross-sectional time-series tobit regressions.

DV: Technical Efficiency Scores	Model 1		Model 2		Model 3	
	Coefficient	SE	Coefficient	SE	Coefficient	SE
Veterans Choice Act (Overall)	−0.164 ***	0.036	-	-	-	-
Veterans Choice Act in 2015	-	-	−0.193 ***	0.052	-	-
Veterans Choice Act in 2016	-	-	−0.328 ***	0.070	-	-
Veterans Choice Act in 2017	-	-	−0.261 **	0.088	-	-
Veterans Using Choice Programs (%)	-	-	−	−	−0.005 **	0.002
COTH Membership	0.013	0.024	0.015	0.023	0.027	0.030
Complexity Level 2	0.089 **	0.039	0.086 **	0.039	0.116 ***	0.043
Complexity Level 3	0.062 *	0.035	0.059 *	0.035	0.074 *	0.040
Hospital Beds	0.00002	0.00006	−0.00001	0.00006	0.00009	0.00006
Veteran Residents in County (1000 s)	0.0003	0.0002	0.0003	0.0002	0.0003	0.0003
Veterans with Bachelor or Above in County (%)	−0.001	0.001	−0.0007	0.001	−0.0004	0.0016
Veteran Poverty Rate in County (%)	−0.002	0.003	−0.003	0.003	−0.0001	0.0032
Veteran Unemployment Rate in County (%)	0.0001	0.002	0.0005	0.002	0.001	0.002
Veterans with Any Disability in County (%)	0.0001	0.002	−0.0002	0.002	−0.00002	0.0022
Medicaid Expansion State	0.016	0.022	0.020	0.021	0.016	0.023
Year (Ordinal Scales by Year (1–6))	0.032 ***	0.009	0.059 **	0.019	0.051 ***	0.016
Constant	0.784 ***	0.082	0.748 ***	0.081	0.502 ***	0.126
Observations	594		594		341	
Log Likelihood	51.976		71.952		35.69	
Wald $\chi^{2}$	36.85 ***		80.55 ***		22.32 **	

* *p* < 0.10, ** *p* < 0.05, *** *p* < 0.01.

## Data Availability

The data that support the findings of this study are available from the American Hospital Association (AHA), U.S. Census Bureau, and Veterans Health Administration (VHA) but restrictions may apply to the availability of these data. Data are, however, available from the authors upon reasonable request and with permission of the AHA and the VHA.
